# DNA barcoding of British mosquitoes (Diptera, Culicidae) to support species identification, discovery of cryptic genetic diversity and monitoring invasive species

**DOI:** 10.3897/zookeys.832.32257

**Published:** 2019-03-19

**Authors:** Luis M. Hernández-Triana, Victor A. Brugman, Nadya I. Nikolova, Ignacio Ruiz-Arrondo, Elsa Barrero, Leigh Thorne, Mar Fernández de Marco, Andreas Krüger, Sarah Lumley, Nicholas Johnson, Anthony R. Fooks

**Affiliations:** 1 Animal and Plant Health Agency, Woodham Lane, Addlestone, Surrey, KT15 3NB, UK; 2 Vecotech Ltd., Keppel Street, London, WC1E 7HT, UK; 3 Department of Disease Control, Faculty of Infectious and Tropical Diseases, London School of Hygiene and Tropical Medicine, Keppel Street, London, WC1E 7HT, UK; 4 Biodiversity Institute of Ontario, University of Guelph, Ontario N1G 2W1, Canada; 5 Center for Rickettsiosis and Arthropod-Borne Diseases, CIBIR, Logroño, La Rioja, Spain; 6 Bunderswehr Hospital Hamburg, Bernhard-Nocht-Str. 74, D-20359 Hamburg, Germany; 7 Public Health England, Porton Down, Salisbury, UK; 8 Faculty of Health and Medical Science, University of Surrey, Guildford, Surrey, GU2 7XH, UK; 9 Department of Clinical Infection, Microbiology and Immunology, Institute of Infection and Global Health, University of Liverpool, L7 3EA, UK

**Keywords:** DNA extraction methods, hidden genetic diversity, molecular identification, vector species

## Abstract

Correct mosquito species identification is essential for mosquito and disease control programs. However, this is complicated by the difficulties in morphologically identifying some mosquito species. In this study, variation of a partial sequence of the cytochrome *c* oxidase unit I (*COI*) gene was used for the molecular identification of British mosquito species and to facilitate the discovery of cryptic diversity, and monitoring invasive species. Three DNA extraction methods were compared to obtain DNA barcodes from adult specimens. In total, we analyzed 42 species belonging to the genera *Aedes* Meigen, 1818 (21 species), *Anopheles* Meigen, 1818 (7 species), *Coquillettidia* Theobald, 1904 (1 species), *Culex* Linnaeus, 1758 (6 species), *Culiseta* Felt, 1904 (7 species), and *Orthopodomyia* Theobald, 1904 (1 species). Intraspecific genetic divergence ranged from 0% to 5.4%, while higher interspecific divergences were identified between *Aedesgeminus* Peus, 1971/*Culisetalitorea* (Shute, 1928) (24.6%) and *Ae.geminus*/*An.plumbeus* Stephens, 1828 (22.5%). Taxonomic discrepancy was shown between *An.daciae* Linton, Nicolescu & Harbach, 2004 and *An.messeae* Falleroni, 1828 indicating the poor resolution of the *COI* DNA barcoding region in separating these taxa. Other species such as *Ae.cantans* (Meigen, 1818)/*Ae.annulipes* (Meigen, 1830) showed similar discrepancies indicating some limitation of this genetic marker to identify certain mosquito species. The combination of morphology and DNA barcoding is an effective approach for the identification of British mosquitoes, for invasive mosquitoes posing a threat to the UK, and for the detection of hidden diversity within species groups.

## Introduction

The family Culicidae includes approximately 112 genera and 3,547 described species (Harbach 2017, 2018). Several species are biting pests playing an important role as vectors of pathogens of humans and livestock (Becker et al. 2010). These include chikungunya, dengue, Japanese encephalitis, yellow fever, West Nile, Rift Valley fever, and Zika viruses, as well as several nematodes and protozoans such as *Plasmodium* Marchiafava & Celli, 1885 (Becker et al. 2010, Medlock et al. 2007). In addition to their medical and veterinary importance, mosquitoes are significant nuisance biters of humans and within the environment may serve additional roles such as key indicators of landscape degradation (Dorvillé 1996, Guedes and Navarro-Silva 2014, Montagner et al. 2018). As a result, mosquitoes are one of the principal target groups within surveillance and control programs worldwide (Hernández-Triana et al. 2017).

Current approaches to species identification still rely heavily upon morphology-based procedures, which typically require substantial training and may not always provide a good resolution on a specimen’s identity due to homogeneity between life stages of different species and the presence of species complexes (Cywinska et al. 2006, Hernández-Triana et al. 2012, 2014, 2015, Linton et al. 2005, Packer et al. 2009, Versteirt et al. 2015). To overcome this obstacle, a small region (658 bp) of the mitochondrial cytochrome *c* oxidase unit I (*COI*) gene was proposed as a standardized DNA marker in support of species identification for animal barcodes, in a process commonly referred to as DNA barcoding (Hebert et al. 2003a, b).

Until recently, thirty-four mosquito species have been recorded in the United Kingdom (UK) (Medlock et al. 2015, Medlock and Vaux 2009, 2015). However, Medlock et al. (2017a) detected the presence of *Ae.albopictus* (Skuse, 1895) in southern England, and Dallimore et al. (2017) collected a single male *Ae.aegypti* (Linnaeus, 1762) in Merseyside in north west England, although these two invasive species are not believed to be locally established. Nonetheless, these findings demonstrated that the UK is at risk of introduction by invasive species of *Aedes* (Dallimore et al., 2017). In addition, Harbach et al. (2017) discovered *Ae.nigrinus* (Eckstein, 1918) in the New Forest, southern England, which brings the total count of named species to 37 [35 native species plus two records of invasive species]. In addition, the occurrence of certain species has been very sporadic as in the case of *Ae.vexans* (Meigen, 1830); however, Medlock et al. (2017b) reported a resident population of this species at Marston Marshes, Norwich. Although no mosquito-borne pathogen affecting humans or livestock is presently thought to circulate in the UK, there is potential for future pathogen emergence (Medlock and Leach 2015; Medlock et al. 2017a, b; Vaux et al. 2015) and there remains continuing mosquito nuisance in various parts of the country (Brugman et al. 2017a, b). Collectively, these discoveries highlight the need for continued research on the native mosquito fauna of the UK, taking into account the potential incursion of invasive species.

There is, however, a paucity of data on the utility of molecular methods for species identification of the British mosquito fauna. During the first development of a molecular assay for the identification of hybrids and sibling species within *Culexpipiens* s.l., Smith and Fonseca (2004) used specimens from England and Scotland. Golding et al. (2012) subsequently employed the *COI* marker to compare sequences of *Cx.modestus* Ficalbi, 1890 with other *Culex* Linnaeus, 1758 species in southeast England, and Danabalan et al. (2012) employed a combination of the internal transcribed spacer gene-2 (*ITS-2*) and *COI* markers in their assessment of molecular identification tools to determine the status of *Cx.pipiens* s.l. The same approach was used by Danabalan et al. (2014) to confirm the occurrence of species within the *Anophelesmaculipennis* complex Theobald, 1911 in England and Wales. Recently, Hernández-Triana et al. (2015) employed an integrated approach to determine mosquito host feeding preferences (via identification of blood meal origin), as well as the molecular characterization of mosquito species carrying pathogens such as myxoma virus (Brugman et al. 2015, 2017a, b) and *Theileriaorientalis* Yakimoff & Soudatschenkoff, 1931 within their bloodmeal (de Marco et al. 2016).

In the present paper, we apply the *COI* DNA barcoding approach in support of the identification of native British mosquitoes and known invasive species in continental Europe. In addition, we assessed the DNA barcode variability using genetic distance methods to detect cryptic diversity across the taxa.

## Materials and methods

### Collection of specimens

**Figure 1.**
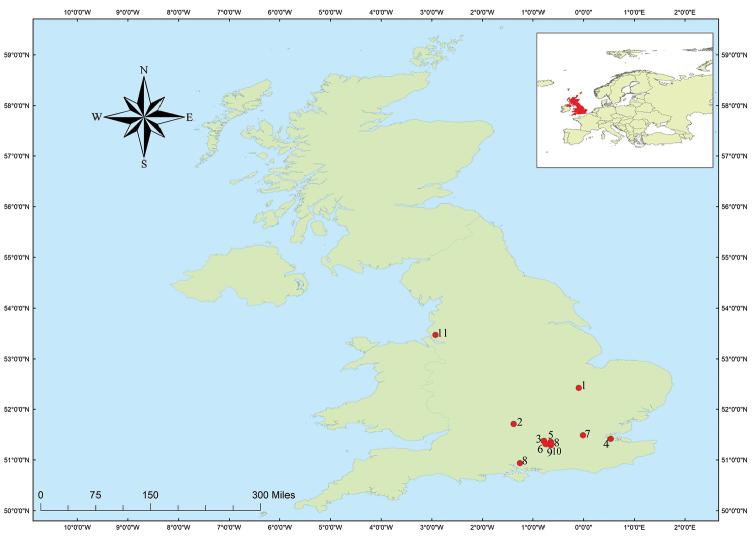
Location of study sites in the United Kingdom. Key: **1** ADAS Arthur Rickwood; **2** Church Farm; **3** Coombelands Farms; **4** Elmley Nature Reserve; **5** Glendell Livery, Mill Lane; **6** Frimley; **7** Mudchute Farm; **8** Northney Farm, Hayling Island; **9** White Lodge, Bisley; **10** Bartley Heath; **11** Dee Marsh.

Ten locations were visited between March and October in the years 2012 to 2015 and specimens were collected following the protocols of Brugman et al. (2015, 2017a, b) (see Table 1, Fig. 1). Further samples were obtained by collecting mosquitoes alighting on the collectors and by standard larval dipping procedures followed by laboratory rearing according to Brugman et al. (2015, 2017a, b). All specimens were kept either at -20 °C or dry-pinned, and were morphologically identified using the key of Cranston et al. (1987). We followed the classification of Wilkerson et al. (2015) for taxa in Aedini. The subgeneric placement for all species can be found in Harbach (2017) and Harbach et al. (2017).

The source of specimens from invasive species is as follows: *Ae.albopictus* – Luke Alphey, UK (colony from Malaysia); Aleksandra Ignjatović-Ćupina, Serbia (wild caught); *Ae.aegypti* – Shahida Begum, UK (colony from West Africa); *Ae.atropalpus* (Coquillet, 1902), *Ae.japonicus* (Theobald, 1901), *Ae.koreicus* (Edwards, 1917) – Norbert Becker and Daniel Hoffman, Germany, and Ignacio Justicia-Ibáñez, Holland (all wild caught); *Culextritaeniorhynchus* Giles, 1901, Filiz Gunay, Turkey (wild caught); *Cx.quinquefasciatus* Say, 1823 [for sequences from NCBI and further details Suppl. material 1].

**Table 1.** Description of key collecting sites with reference to habitat and the main livestock present. Further information can be found in Brugman et al. (2017b).

Locality/Farms	County	Coordinates	Habitat	Main livestock types present
1. ADAS Arthur Rickwood	Cambridgeshire	52.422560, -0.098302	Grazing farm	Sheep
2. Church Farm	Oxfordshire	51.715807, -1.380813	Rural area	Cattle, sheep
3. Coombelands Farm	Surrey	51.360241, -0.652256	Mixed farm	Cattle, sheep, pigs, horses
4. Elmley Nature Reserve	Kent	51.377587, 0.783954	Grazing marsh	Cattle, sheep
5. Glendell Livery, Mill Lane	Surrey	51.290499, -0.652256	Mixed woodland	Horses
6. Frimley	Surrey	51.313037, -0.745237	Peri-urban	n/a
7. Mudchute Farm	Greater London	51.491732, -0.009367	City farm	Cattle, sheep, pigs, horses
8. Northney Farm, Hayling Island	Hampshire	50.828166, -0.962151	Arable farm	Cattle
9. White Lodge, Bisley	Surrey	51.322255, -0.637692	Mixed woodland	Cattle
10. Bartley Heath	Hampshire	50.919701, -1.565337	Woodland	Cattle, horses, deer
11. Dee Marsh	Cheshire	52.8322, -3.7656	Salt marsh	n/a

### DNA extraction methods

Three methods were used for DNA extraction from two mosquito tissue types (Brugman et al. 2015, 2017). Firstly, 1–2 legs of specimens were placed in 100 µl of molecular grade water in a 96-well plate, which was then sonicated at room temperature for 10 min to release DNA (Hunter et al. 2008). Secondly, we employed a modified Hotshot technique (Montero-Pau et al. 2008). In this case, 1–2 legs were placed directly into 50 µl of alkaline lysis buffer in a 96-well plate, which was then sonicated in a water bath for 10 min. The plate was subsequently incubated in a thermocycler for 30 min at 94 °C, cooled for 5 min at 4 °C, and then centrifuged for 3 min at 3000 rpm, after which 50 µl of the neutralizing buffer was added to each sample. The plate was then centrifuged again for 10 min at 3000 rpm, and stored at -80 °C until analysis. Thirdly, engorged female abdomens were processed using Qiagen DNeasy Blood and Tissue kits following the procedures detailed in Brugman et al. (2015, 2017a, b) and the manufacturer’s instructions.

### COI DNA barcoding region amplification

For molecular species identification using the *COI* DNA barcoding region, the protocols of Hernández-Triana et al. (2012, 2014) and Hebert et al. (2003a, b) were followed. We used the primers developed by Folmer et al. (1994), which amplify the 658 bp long target region of the *COI* gene. PCR products were obtained using a Qiagen PCR system following the reaction mix of Hernández-Triana et al. (2017).

### Data analysis

**Figure 2.**
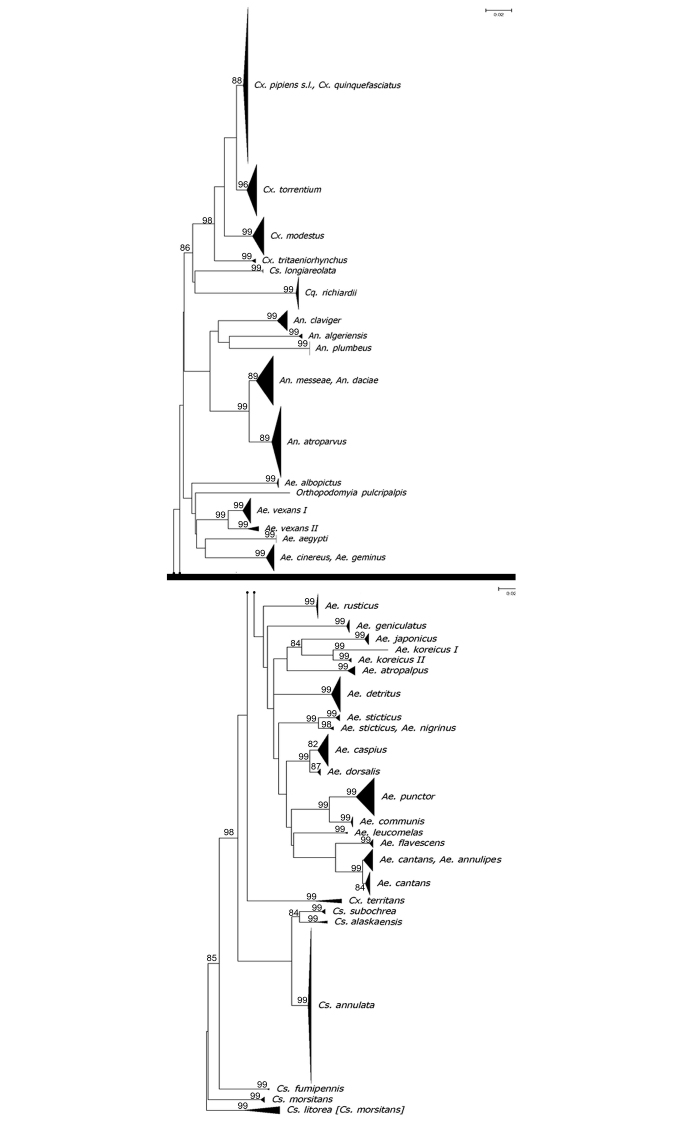
Neighbor joining tree of *COI* DNA barcodes (658 bp) for mosquito species. A divergence of > 2% may be indicative of separate operational taxonomic units. Only bootstrap values higher than 70% are shown.

Paired bi-directional sequence traces were combined to produce a single consensus sequence (i.e., the full-length 658 bp barcode sequence). To achieve this, individual forward and reverse traces were oriented, edited, and aligned using the Sequencer (v.4.5; Genes Codes Corporation, Ann Harbour, MI), GenDoc (v. 2.6.02) and ClustalX sequence analysis programs (Hernández-Triana et al. 2017). Full details for each specimen and sequence information can be found at the Barcode of Life Database (BOLD) within the “Human Pathogens and Zoonoses Initiative”, Working Group 1.4. The Digital Object Identifier (DOI) for the publically available projects in BOLD is dx.doi.org/10.5883/DS-MQFWUK and dx.doi.org/10.5883/DS-MQIUV. Accession numbers for all sequences were obtained from NCBI (accession numbers: MK403007-MK403548). For certain species, we used *COI* barcode sequences deposited at NCBI due to the lack of available material from UK populations (Table 3; Suppl. material 1). The dataset was analyzed in MEGA v.6 (Tamura et al. 2007). The Neighbor Joining (NJ) analysis was performed using the Kimura 2-Parameter distance metric to determine their distribution pattern. The tree robustness was measured by the bootstrap approach using 1000 pseudoreplicates (Hernández-Triana et al. 2012, 2014). To barcode sequences larger than 500 bp, a Barcode Index Number (BIN) was assigned and each BIN was mapped according to species (see Fig. 2). The taxonomic discordance in the dataset (Hernández-Triana et al. 2017) was analyzed using BOLD as detailed in Ratnasingham and Hebert (2013).

## Results

### Assessment of DNA extraction methodologies

In general, adding 1–2 legs to molecular grade water and then sonicating them for 10 min proved to be an effective method for obtaining DNA (30 min total time); however, only 41 barcodes (43.1%) yielded sufficient sequence data for inclusion in our analysis (Table 2). The Hotshot technique also proved to be an efficient approach (1 hour per plate) for processing 1–2 legs with high percentages of target DNA amplification and *COI* DNA barcode sequences (429 sequences, 90.3%). In terms of cost, reagents for the preparation of the Hotshot working stock buffers were only 200 GBP, one purchase of which we estimate can last up to one year. DNA extraction from blood-engorged abdomens processed using the Qiagen DNeasy Blood and Tissue kit also provided barcodes for 306 specimens (64.4%), but this approach was time consuming, with a sample processing rate of 32 specimens per four hour session of DNA extraction. The time for the DNA extraction was also increased by the limitation of the number of wells in the centrifuge available (30 wells). In addition, non-target PCR product was also encountered as vertebrate DNA present was amplified from cows, chicken, sheep, rabbits and birds [169 samples] (see Table 2).

**Table 2.** DNA extraction methods and percentage of PCR amplification success in obtaining *COI* DNA barcodes from mosquitoes.

Extraction method	No. plates / samples	Time per plate	Amplification success n (%)	Observations
1. Legs directly into molecular grade water and sonicated for 10 min	1 plate / 95 samples	30 min	41 (43.1%)	High sequencing failure (54 samples)
2. Legs directly into alkaline lysis buffer and sonicated for 10 min (Hotshot)	5 plates / 475 samples	1hr each plate	429 (90.3%)	Target length barcodes obtained
3. Abdomen processed using Qiagen kit	5 plates / 475 samples	Only 32 samples per 4hr session for DNA extraction for each plate	306 (64.4%)	Target length barcodes obtained. Vertebrate DNA amplified

### Mosquito species identification using DNA barcoding

In total, we analyzed DNA barcode sequences for 42 species belonging to the genera *Aedes* (21 species), *Anopheles* (7 species), *Coquillettidia* (1 species), *Culex* (6 species), *Culiseta* (7 species), and *Orthopodomyia* (1 species) (Table 3). Of these, we analyzed sequences for 23 of the 37 species of mosquito that have been recorded in the UK (62%) (Harbach et al. 2017, Medlock et al. 2007a, b). In addition, we also analyzed representatives of invasive *Aedes* species (*Ae.aegypti*, *Ae.albopictus*, *Ae.japonicus*, *Ae.koreicus*, *Ae.atropalpus*) and two *Culex* species (*Cx.quinquefasciatus*, *Cx.tritaeniorhynchus*), which are of epidemiological relevance in Europe (Medlock et al. 2017a, b). Three or more representatives were available for 38 morphospecies in the dataset (see Table 3). In total, 1198 barcode sequences were analyzed.

Even though in most cases individuals of the same species clustered together, this was not the case for all species. Within the genus *Aedes*, the first incongruence was identified between *Ae.sticticus* (Meigen, 1838) and *Ae.nigrinus*. Although the majority of specimens from Belgium and the two UK specimens identified as *Ae.sticticus* (voucher number APHA-4-2015G06, APHA-4-2015G07) grouped together in a separate cluster with 100% bootstrap support, the only two available *COI* sequences of *Ae.nigrinus* in NCBI (KP942769, KP942770) grouped with the two specimens collected in Belgium, identified as *Ae.sticticus* (CULBE-833009, CULBE-833008) (Fig. 2). To further support our identification of the two UK specimens as *Ae.sticticus*, we obtained *ITS-2* sequences (data not shown) and searched the NCBI database using the BLAST algorithm; both queries retrieved *Ae.sticticus* with 96% match (KF535079) [this relative low percentage could be due to the low coverage of the *ITS-2* sequences we obtained (338 bp and 369 bp, respectively)]. Similar results have been obtained by Versteirt et al. (2015) (see Fig. 2). Certain specimens grouped only as *Ae.cantans* or *Ae.annulipes*, but another group was composed of *Ae.cantans* and *Ae.annulipes* (Fig. 1) with 100% bootstraps support values. Similarly, no successful identification was reached between *Ae.cinereus* (Meigen, 1818) and *Ae.geminus*, species which are morphologically similar.

Within *Anophelesmaculipennis* s.l. (Linton et al. 2002, 2005), no accurate identification was achieved between *An.messeae* [also molecularly identified by *ITS-2* in our laboratory; see also Kronefeld et al. 2012] and *An.daciae* (sensu Nicolescu et al. 2004), although *An.atroparvus* was clearly separated from the aforementioned species (Fig. 2). This is not surprising as all members of the *An.maculipennis* complex are phylogenetically related, and cannot be readily identified based on adult morphological traits or only using the *COI* genetic marker (Linton et al. 2002, Kronefeld et al. 2014, Ruiz-Arrondo et al. 2017). In the genus *Culex*, *COI* was not able to separate *Culexpipiens* s.l. (including both forms *pipiens* and *molestus*) and *Cx.quinquefasciatus*, in agreement with results by Gunay et al. (2015).

Our DNA barcodes dataset from the genus *Culiseta* separated certain species with high support bootstrap values such as *Cs.alaskaensis* (Ludlow, 1906), *Cs.annulata* (Schrank, 1776), *Cs.longiareolata* (Macquart, 1838) and *Cs.subochrea* (Edwards, 1921) (Fig. 2). However, we could not achieve the same resolution for *Cs.fumipennis* (Stephens, 1825), *Cs.litorea* (Shute, 1928) and *Cs.morsitans* (Theobald, 1901). The specimens identified as *Cs.fumipennis* (Versteirt et al. 2015) from Belgium grouped separately from one specimen (KU748471) collected in the UK (de Marco et al. 2016) previously identified as *Cs.fumipennis*, which clustered with specimens from Belgium identified as *Cs.morsitans* (CULBE-816017, CULBE-816018, CULBE-997001, CULBE-997002, CULBE-9972101, CULBE-972103) with 99% bootstrap values. Therefore, we now consider the UK specimen to be *Cs.morsitans*. In addition, seven specimens from the UK identified as *Cs.morsitans* in de Marco et al. (2016) (KU748440, KU748443, KU748450, KU748453, KU748460, KU748500, KU748488), grouped with 99% bootstraps values with two males recently collected from Spain identified as *Cs.litorea*. Subsequent dissection of the genitalia of these specimens confirmed their identification as *Cs.litorea* based on the key of Becker et al. (2010); therefore, we now considered these seven specimens from the UK as *Cs.litorea*.

The levels of sequence divergence were variable across the taxa, with conspecific individuals collected from a single site often exhibiting zero, or 0.07% to 0.1% divergence values, while other specimens showed higher percentages (see Table 3). The intraspecific genetic divergence measured 1.3%, ranging from 0% to 5.4% (Table 3) (*Ae.aegypti*, *An.plumbeus*, *Cx.modestus* and *Cx.quinquefasciatus*), while the interspecific divergence ranged between 0.19% to 24.6% (Suppl. material 2). Interspecific genetic divergence values were higher between species from different genera. The pairs *Ae.geminus/Cs.litorea* (24.6%) and *Ae.geminus*/*An.plumbeus* (22.5%) were the most divergent species. As known, the smallest values of genetic divergence were found among species in the same genus, for example *Cx.pipiens* s.l./*Cxquinquefasciatus* (0.19%), *Ae.cantans*/*Ae.annulipes* (1.2%) and *Ae.geminus*/*Ae.cinereus* (0.65%) (Suppl. material 2).

**Table 3.** List of mosquito species (in alphabetical order), country of collection, and number of specimens with DNA barcodes. Mean (%) intraspecific values of sequence divergence (Kimura2-Parameter distance) are shown with missing entries indicating that less than two specimens were analyzed. Asterisks indicate species complexes (*) and taxa with deep splits (**) in the Neighbor Joining tree; (***) taxa with above 2% genetic divergence. Invasive species in Europe are in Bold.

Species	Collection Country	*n*	mean %
*** Aedes aegypti ***	West Africa	10	0
*** Aedes albopictus ***	Malaysia; Montenegro	12	0.12
* Aedes annulipes *	Belgium	12	0.89
*** Aedes atropalpus ***	Holland, USA, Canada	11	0.69
* Aedes cantans *	Belgium; UK	44	0.80
* Aedes caspius *	Belgium; UK	40	0.78
* Aedes cinereus *	Sweden; UK	30	0.61
* Aedes communis *	Belgium	13	0.14
* Aedes detritus *	Belgium; UK	44	0.66
* Aedes dorsalis *	USA; Canada	8	0.16
* Aedes flavescens *	UK	10	0.18
* Aedes geminus *	Germany	4	0.58
* Aedes geniculatuss *	Belgium	16	0.25
*** Aedes japonicus ***	Germany	14	0.32
Aedeskoreicus**^;^***	Belgium; Holland; Hungary	6	2.19
* Aedes leucomelas *	Sweden	2	0.40
* Aedes nigrinus *	Sweden	2	0.77
* Aedes punctor *	Belgium; UK	47	0.67
* Aedes rusticus *	Belgium; UK	31	0.07
* Aedes sticticus *	Belgium; UK	10	1.29
Aedesvexans**	Belgium; Spain; Holland; Sweden; UK	38	1.46
* Anopheles algeriensis *	Spain	6	0.41
* Anopheles atroparvus *	UK; Belgium	91	0.92
*Anophelesclaviger* s.l.	Belgium; UK	26	0.65
* Anopheles daciae *	Romania; UK	28	0.76
* Anopheles messeae *	UK	35	1.01
* Anopheles plumbeus *	Belgium; UK	17	0
* Coquillettidia richiardii *	Belgium; UK	42	0.07
* Culex modestus *	Germany; Romania; Turkey; UK	49	0
*Culexpipiens* s.l.*	Belgium; UK	187	0.06
*** Culex quinquefasciatus ***	Pakistan; Turkey	12	0
* Culex territans *	Belgium; Germany	5	2.05
* Culex torrentium *	Belgium; Germany; UK	66	0.43
*** Culex tritaeniorhynchus ***	Turkey	5	0.65
* Culiseta alaskaensis *	Canada	3	1.13
* Culiseta annulata *	Belgium; UK	192	0.05
* Culiseta fumipennis *	Belgium	2	0.30
Culisetalitorea***	Spain; UK	9	5.35
* Culiseta longiareolata *	Spain	5	0.12
* Culiseta morsitans *	Belgium; UK	7	0.34
* Culiseta subochrea *	Spain; UK	6	0.34
* Orthopodomyia pulcripalpis *	Austria	1	n/a

In this study, we analyzed three species which are known, or suspected to be, part of species complexes [species which can only be distinguished either by cytotaxonomy or molecular methods (Danabalan et al. 2012, 2014; Linton et al. 2001)]: *An.maculipennis* s.l., *An.claviger* s.l. (Meigen, 1804), and *Cx.pipiens* s.l. All specimens grouped together in either *Cx.pipiens* s.l. or *An.claviger* s.l., and we did not detect high levels of genetic diversity or deep splits in the NJ tree as found in other studies (Gunay et al. 2015, Versteirt et al. 2015). This might be due to specimens originating from localities in relatively close proximity to one another in England (Fig. 1). Specimens of *Cx.pipiens* s.l. in this study originated from the study of Brugman et al. (2015, 2017), in which the *CQ11* PCR assay was conducted to separate the forms *molestus* and *pipiens*. Only specimens from the typical *pipiens* form of *Cx.pipiens* were detected in the aforementioned studies, with 0.06% genetic diversity in our dataset. Nonetheless, not all morphologically identified species clustered as expected. Certain species exhibited higher levels of divergence above 2% (see Table 1) and other showed deep splits in the NJ tree (Fig. 2). For example, intraspecific genetic divergence averaged 1.46% in *Ae.vexans*, but the specimens separated into two defined clusters (I and II) (Fig. 2). Similarly, *Ae.koreicus* showed a deep split in the NJ with 2.19% genetic divergence.

The BIN counts in our dataset in BOLD of 721 full length barcode sequences from 1006 records in BOLD datasets found 21 BINs. The BIN analysis did not include sequences downloaded from the NCBI database. In general, 487 barcodes were assigned a BIN number, which represented 14 concordant BINs, three BINs were singletons (*Cs.fumipennis*BOLD:AAR2210, *Ae.geniculatus*BOLD:AAM5898, and *Cs.subochrea*BOLD:AAV90 75), and only four BINs (231 records) were discordant. The discordant BINs occurred at the species level, mainly because of the discrepancy in taxonomic information assigned to certain specimens, for example *Ae.cinereus* versus Ae.nr.cinereus, and *Ae.caspius* versus Ae.nr.caspius. Another discordance was in a single specimen identified as *Cx.torrentium*, which appears to be close to a BIN within *Cx.pipiens* s.l. (BOLD:AAA4751; Process ID:MSEMV855-15); however, morphological traits in the male genitalia and other analysis (*CQ11* PCR) showed that it does belongs to *Cx.torrentium* (Manley et al. 2015), and it did cluster with this species when further 66 barcode sequences of *Cx.torrentium* were added to the dataset.

## Discussion

This study assessed minimally destructive approaches that retained a significant part of the sample as a referenced voucher and the development of a *COI* DNA barcoding library in mosquitoes, and assessed the use of the variability within *COI* in support of species identification. Overall, the three extraction methods used provided sufficient DNA for subsequent analysis; however the modified Hotshot technique of Montero-Pau et al. (2008) proved to be the most efficient and inexpensive method for obtaining *COI* DNA barcode sequences. This method has been applied to other groups such as the Hymenoptera (Guzmán-Larralde et al. 2016) with good results as assessed by DNA yield and PCR amplification success. The amplification of vertebrate DNA from engorged abdomens using the Qiagen DNeasy Blood and Tissue extraction kit highlights the need to use insect specific primers, for example LepF/LepR (see www.boldsystems.org/index.php/ Public_Primer_PrimerSeach) instead of the standard Folmer primers (Folmer et al. 1994). In terms of cost, considering that we did not have to buy a DNA extraction kit to perform the DNA extraction for processing the legs, the Hotshot technique represented savings of around 500 GBP per 96-well plate to our laboratory, making it a cost-effective method for performing DNA extractions.

The majority of morphologically identified species in this study formed defined groups in the NJ analysis based on DNA barcodes (Fig. 2), supporting the use of *COI* DNA barcoding in combination with morphological observation as a suitable approach for species identification. Genetic divergence between morphospecies ranged from 0.19% to 24.6%, whereas intraspecific genetic divergences within distinct species ranged from 0% to 5.4% (average 1.30%; Table 1, Suppl. material 2). Most of the specimens within a morphospecies were resolved in the NJ tree (Fig. 2). However, some individuals in certain taxa such as *Ae.annulipes*/*Ae.cantans*, *Ae.cinereus*/*Ae.geminus*, *Ae.sticticus*/*Ae.nigrinus*, *An.daciae*/*An.messeae*, and *Cx.quinquefasciatus*/*Cx.pipiens* s.l. clustered together (Fig. 2), indicating some limitations of the *COI* gene as a marker to separate these species. This finding is not surprising as these taxa are phylogenetically, as it has been highlighted in the literature (Harbach 2017, Harbach et al. 2017).

With regard to *Anophelesmaculipennis* s.l., although some morphological traits in egg structure provide an effective method to separate some members of this group, there is some dispute regarding the taxonomic status of *An.daciae* (e.g. Linton et al. 2002, 2005, Kronefeld et al. 2012). This species was described by Nicolescu et al. (2004) based on all life stages collected from the Black Sea region in Romania. The authors stated that *An.daciae* and *An.messeae* have been misidentified in the past because of similar morphology. However, they showed that *An.daciae* eggs are generally smaller than those of *An.messeae*, with patches of larger deck tubercles that contrast more sharply with patches of smaller tubercles to impart greater definition to the mottled surface of the deck (see Nicolescu et al. 2004; fig. 3 A, C). In contrast, the deck of *An.messeae* eggs has a more diffuse or weakly mottled appearance (see Nicolescu et al. 2004; fig. 3 B, D). In the same study, molecular analysis of *ITS-2* sequences identified single nucleotide polymorphisms and unique haplotypes which, in the authors’ views, confirmed the specific status of *An.daciae*. However, other authors have queried the specific status of *An.daciae*, and stated that *COI* offered poor resolution and advocated for further work to determine the status of *An.daciae*. Similarly, in their study of Belgian mosquitoes, Versteirt et al. (2015) reported that specimens identified as *Ae.annulipes* and *Ae.cantans* grouped together in their NJ tree, and stated that *COI* cannot separate these two species; the same results have been obtained in our study (Fig. 2).

Moreover, *COI* DNA barcoding highlighted mis-identifications within the genus *Culiseta* (*Cs.fumipennis*, *Cs.litorea* and *Cs.morsitans*). These species are placed in the subgenus Culicella, and the females are difficult to identify because of their morphological similarity and absence of reliable diagnostic characteristics (Becker 2010, Cranston et al. 1987, Medlock and Vaux 2010). All of our females were collected in traps and were in relatively poor condition. In addition, no *COI* sequences of reliably identified material of *Cs.litorea* were available in the NCBI or BOLD databases to compare with our specimens (Ruiz-Arrondo et al. 2017). Because the sequences of females of *Cs.morsitans* matched the two males identified as *Cs.litorea* from Spain, we considered all our specimens to be *Cs.litorea*. In the literature (Medlock et al. 2007), *Cs.morsitans* is considered a widespread species in the UK, found in permanent waters, while *Cs.litorea* has a more restricted distribution in coastal regions of southern England. Both species feed mainly on birds, but can also bite humans, and they are considered bridge vectors of arboviruses (Medlock et al. 2007). Our findings highlight the need for careful examination of material obtained from traps in combination with the application of molecular techniques for a reliable identification of these species. Even though in our dataset *Ae.vexans* showed a low genetic divergence of approximately 2%, specimens of this species separated into two distinct groups in the NJ tree (Fig. 2), which may be an indication of some genetic differentiation within the population. This agrees with the findings of Lilja et al. (2018) in which the authors reported a distinct genotype of *Ae.vexans* in Europe.

Regarding non-indigenous mosquito species, although the adults of certain species are easily identified using morphological keys, for example *Ae.aegypti* and *Ae.albopictus* (Becker 2010, Cranston et al. 1987), the development of a molecular library for species identification is important, in particular when specimens are found in a poor stage of preservation. This is an essential step for the establishment of control measures (Versteirt et al. 2015) in the event of a recent introduction, as in the case of the detection of *Ae.albopictus* in the UK (see Medlock et al. 2017a, b).

In our dataset, *Ae.koreicus* and *Cs.litorea* showed higher intraspecific genetic divergences (Table 1, Fig. 2), which may indicate the presence of cryptic diversity. For all other species, the variation in intra- and interspecific genetic values reported in this study fall within the range for DNA barcoding studies of European mosquitoes (Engdahl et al. 2014, Gunay et al. 2015, Versteirt et al. 2015) or other zoogeographical regions such as the Nearctic and Oriental Regions (Cywinska et al. 2006, Kumar et al. 2007, Murugan et al. 2016). Nonetheless, we advocate the combination of the *COI* DNA barcoding with other genetic markers such as the Elongator Complex Protein 1 gene (*ECP1*) (Low et al. 2016, Pangjanda and Pramual 2016, Senatore et al. 2014) and *ITS-2* sequences from a larger number of specimens across the species distribution range in order to resolve some of the taxonomic problems highlighted in this study.

## Conclusions

This study provides *COI* DNA barcoding data to support the molecular identification of mosquito species in the UK as well as invasive mosquito species, many of which are currently expanding their geographical range in continental Europe. We augment the barcoding data for anthropophilic species such as *Ae.cinereus*, *Ae.detritus*, *Ae.sticticus*, *Ae.vexans*, and *Cx.modestus*, as well as other species of veterinary importance such as the bridge vector *Cs.annulata*. Even though the majority of specimens were separated by *COI*, certain taxa could not be distinguished using this genetic marker within the genera *Aedes*, *Anopheles* and *Culex*. The use of *COI* also underlined identification problems in *Culiseta* species (*Cs.fumipennis*, *Cs.litorea* and *Cs.morsitans*) within the BOLD and NCBI databases. This finding supports the need for continuing research combining the use of molecular methodologies with morphological traits for species delineation in the Culicidae.

## Supplementary materials

Supplementary material 1 Accession number(s) of COI DNA barcode sequences used in this study downloaded from the NCBI database or provided by colleagues Data type: molecular data This dataset is made available under the Open Database License (http://opendatacommons.org/licenses/odbl/1.0/). The Open Database License (ODbL) is a license agreement intended to allow users to freely share, modify, and use this Dataset while maintaining this same freedom for others, provided that the original source and author(s) are credited.Luis M. Hernández-Triana, Victor A. Brugman, Nadya I. Nikolova, Ignacio Ruiz-Arrondo, Elsa Barrero, Leigh Thorne, Mar Fernández de Marco, Andreas Krüger, Sarah Lumley, Nicholas Johnson, Anthony R. Fooks

Supplementary material 2 Percentage of Interspecific (between groups) pairwise K2P genetic divergence of unique DNA barcodes (658 bp), representing 42 species of mosquitoes Data type: molecular data Explanation note: Highest pairwise distances (most divergent taxa) and lowest pairwise distances (most closely related taxa) are highlighted in yellow/bold, and green, respectively. This dataset is made available under the Open Database License (http://opendatacommons.org/licenses/odbl/1.0/). The Open Database License (ODbL) is a license agreement intended to allow users to freely share, modify, and use this Dataset while maintaining this same freedom for others, provided that the original source and author(s) are credited.Luis M. Hernández-Triana, Victor A. Brugman, Nadya I. Nikolova, Ignacio Ruiz-Arrondo, Elsa Barrero, Leigh Thorne, Mar Fernández de Marco, Andreas Krüger, Sarah Lumley, Nicholas Johnson, Anthony R. Fooks
