# Supplementary material for: DNA barcoding of British mosquitoes (Diptera, Culicidae) to support species identification, discovery of cryptic genetic diversity and monitoring invasive species
Source: Zookeys. 2019 Mar 19;832:57–76. doi: 10.3897/zookeys.832.32257 (PMC6435598; doi:10.3897/zookeys.832.32257)
Supplement: Supplementary material 1 [file zookeys-832-057-s001.docx]

**Additional File S1.** Accession number(s) of COI DNA barcode sequences used in this study downloaded from the NCBI database or provided by colleagues.

*>****Aedes atropalpus***

KM457565, KM457566, KM457567, MG242470, GU907849, GU907848, GU907847, GU907846, GU907845, GU907844

**>*Aedes annulipes***

CULBE-74110, CULBE-971132, CULBE-389014, CULBE-389016, CULBE-968445, CULBE-776009, CULBE-971131, CULBE-971120, CULBE-321009, CULBE-358001, CULBE-186011, CULBE-186012

***>Aedes cantans***

CULBE-968154, CULBE-501003, CULBE-501002, CULBE-358003, CULBE-186019, CULBE- 321008, CULBE-358047, CULBE-186010, CULBE-358056, CULBE-358002, CULBE-776017, CULBE-776012

***>Aedes caspius***

CULBE-969102, CULBE-959010, CULBE-939039, CULBE-969101, CULBE-959012, CULBE-476149, CULBE-476165, CULBE-387004, CULBE-387002, CULBE-575043, CULBE-575044, CULBE-575045

>***Aedes cinereus***

CULBE-358006, CULBE-855008, CULBE-855014, CULBE-789032, CULBE-797002, CULBE-789027, CULBE-855015, CULBE-552018, CULBE-552025, CULBE-552033, CULBE-462009, CULBE-55003, CULBE-462018

***>Aedes communis***

CULBE-865011, CULBE-865003, CULBE-787008, CULBE-787011, CULBE-776048, CULBE-838002, CULBE-852011, CULBE-787012, CULBE-787016, RL2M10021, RL2M10024, RL2M1002

**>*Aedes detritus***

CULBE-52024, CULBE-952030, CULBE-952023, CULBE-951019, CULBE-951030, CULBE-951038, CULBE-952005, CULBE-951039, CULBE-951035

***>Aedes dorsalis***

MG242488, KR684167, KR683985, KR683934, KR683506, KR682920, KR682876, KR682235

**>*Aedes geminus* [provided by Andreas Krüger]**

25_Aedes_geminus_MV, 26_Aedes_geminus_MV, 28_Aedes_geminus_HH, 32_Aedes_geminus_SH

***>Aedes geniculatus***

CULBE-964014, CULBE-976198, CULBE-976196, CULBE-771003, CULBE-771004, CULBE-762001, CULBE-771002, CULBE-964001, CULBE-855001, CULBE-847004, CULBE-847009, CULBE-822008, CULBE-804001, CULBE-804002, CULBE-822007

***>Aedes japonicus***

960149, 960150, Aejap001, Aejap002

*>****Aedes koreicus***

KM457599, KM457600, KT358407, KM258298, KM258299, KX524947

*>****Aedes leucomelas***

KP942730, KP942729

***>Aedes nigrinus***

KP942770, KP942769

***>Aedes punctor***

RL2M10026, RL2M10022, RL2M10028, RL2M10027, RL2M100212, 399093, 787006, RL2M100211, CULBE-787010, CULBE-910013, CULBE-857025, CULBE-881014, CULBE-857021, CULBE-910007, CULBE-910019, CULBE-910005, CULBE-91000, CULBE-881015, CULBE- 910012

***>Aedes rusticus***

CULBE-501004, CULBE-127013l, CULBE-127011, CULBE-186003, CULBE-127014, CULBE-186007, CULBE-186006, CULBE-186002, CULBE-838019, CULBE-838018

**>*Aedes sticticus***

CULBE-966050, CULBE-966061, CULBE-110030, CULBE-853010, CULBE-110017, CULBE-833009, CULBE-399089, CULBE-833008

***>Aedes vexans***

CULBE-966066, CULBE-647010, CULBE-966091, CULBE-966068, CULBE-215021, CULBE-215024, CULBE-215022, CULBE-058018, CULBE-268012, CULBE-058020, CULBE-476048, CULBE-476026, CULBE-476030, CULBE- CULBE-163012, CULBE-163014, CULBE-163018

***>Anopheles claviger***

CULBE-237020, CULBE-455039, CULBE-918003, CULBE-046010, CULBE-389001, CULBE-593307, CULBE-172022, CULBE-593310

**>*Anopheles daciae***

AY757922, AY757923, AY757924, AY757925, AY757926, AY757927, AY757928, AY757929, AY757930, AY757931, AY757932, AY757933, AY757934, AY757935, AY757936, AY757937, AY757938, AY757939, AY757941, AY757945, AY757946, AY757947, AY757948, AY757949, AY757950, AY757951, AY757953, AY757954

***>Anopheles messeae***

CULBE-191245, CULBE-939094, CULBE-018001, CULBE-046003, CULBE-684004, CULBE-046004

***>Anopheles plumbeus***

CULBE-249004, CULBE-249002, CULBE-046009, CULBE-046038, CULBE-046034

**>*Coquillettidia richardii***

CULBE-941022, CULBE-968008, CULBE-968010, CULBE-191010, CULBE-126009, CULBE-191012, CULBE-191011, CULBE-968009, CULBE-009239, CULBE-939018, CULBE-142004, CULBE-009243, CULBE-142005, CULBE-126010, CULBE-126008

>***Culex modestus***

KU175266, KT876488, KT876486, KT876479, KJ401305, KJ401304, KJ401303, KM280578, KM452947, KJ401302, KJ401301, KF754813, KF754812, KF754811, KF754810, KJ012102, HF562837, JN592748, JN592747, JN592746, JN592745, JN592744, JN592743, JN592742, JN592735, JN592734, JN592733, JN592732, JN592731, JN592730, JN592728, JN592727, JN592726, JN592725, JN592724, JN592723, FM177758.

*>****Culex pipiens* s.l.**

CULBE-144001, CULBE-144003, CULBE-144005, UCL004, UCL013, UCL015, CULBE-144007, CULBE-144009, CULBE-144011, CULBE-962006, CULBE-956012, CULBE-615012, CULBE-962039, CULBE-962019, CULBE-959030, CULBE-023030, CULBE-023023, CULBE-023016, CULBE-313015, Rui2, CULBE-857069, CULBE-313011, CULBE-046007, CULBE-615003, CULBE-615008, CULBE-991023, CULBE-991003, CULBE-857020.

*>C****ulex quinquefasciatus***

KF407796, KF407795, KF407794, KF407793, KF407792, KF407791, KF407790, KF407789, KF407788, KF407787, KF407786, KF407785

***>Culex territans***

CULBE-975040, CULBE-287044, CULBE-852013, CULBE-833048

***>Culex torrentium***

CULBE-857130, CULBE-023005, CULBE-215038, CULBE-857039, CULBE-857040, HE997145, HE997146, KJ012235, KJ012236, KJ012237, KJ012238, KJ012239, KJ012240, KJ012241, KJ012242, KJ012242, KM258157, KM258158, KM258159, KM258160, KM258161, JQ253831, JQ253833, JQ253806, JQ253807, JQ253808, JQ253809, JQ253810, JQ253811, JQ253812, JQ253813, JQ253815, JQ253816, JQ253817, JQ253818, JQ253819, JQ253820, HE997146, KM258157, KM258158, KM258159, KM258161, KM258160, KJ012235, KJ012236, KJ012237, KJ012238, KJ012239, KJ012242, KJ012241, KJ012240, HE997142, HE997144, HE997145, JQ253821, JQ253822, JQ253823, JQ253824, JQ253825, JQ253826, JQ253827, JQ253828, JQ253829, JQ253830, JQ253832

***> Culiseta alaskaensis***

KX675391, KU874740, KU874741

***>Culiseta annulata***

CULBE-06030, CULBE-006031, CULBE-939021, CULBE-939042, CULBE-544024, CULBE-939006, CULBE-838046, CULBE-838041, CULBE-838040, CULBE-338085, CULBE-941002, CULBE-130051, CULBE-338084, CULBE-544018, CULBE-544019, CULBE-939043

***>Culiseta fumipennis***

CULBE-876004, CULBE-763007

*>C****uliseta litorea* [provided by Ignacio Ruiz-Arrondo]**

17CM3_8_Clitorea_male, 17CM3_3_Cs_litorea_male

***>Culiseta morsitans***

CULBE-972103, CULBE-997001, CULBE-997002, CULBE-816018, CULBE-816017, CULBE-972102

>***Orthopodomyia_pulcripalpis***

KY608735
